# Data-driven insights can transform women’s reproductive health

**DOI:** 10.1038/s44294-024-00019-x

**Published:** 2024-05-14

**Authors:** Tomiko T. Oskotsky, Ophelia Yin, Umair Khan, Leen Arnaout, Marina Sirota

**Affiliations:** 1 Bakar Computational Health Sciences Institute, University of California, San Francisco, San Francisco, CA USA; 2 Maternal–Fetal Medicine, Department of Obstetrics, Gynecology & Reproductive Sciences, University of California, San Francisco, San Francisco, CA USA; 3 Department of Pediatrics, University of California, San Francisco, San Francisco, CA USA

**Keywords:** Reproductive disorders, Reproductive biology, Reproductive disorders

## Abstract

This perspective explores the transformative potential of data-driven insights to understand and address women’s reproductive health conditions. Historically, clinical studies often excluded women, hindering comprehensive research into conditions such as adverse pregnancy outcomes and endometriosis. Recent advances in technology (e.g., next-generation sequencing techniques, electronic medical records (EMRs), computational power) provide unprecedented opportunities for research in women’s reproductive health. Studies of molecular data, including large-scale meta-analyses, provide valuable insights into conditions like preterm birth and preeclampsia. Moreover, EMRs and other clinical data sources enable researchers to study populations of individuals, uncovering trends and associations in women’s reproductive health conditions. Despite these advancements, challenges such as data completeness, accuracy, and representation persist. We emphasize the importance of holistic approaches, greater inclusion, and refining and expanding on how we leverage data and computational integrative approaches for discoveries so that we can benefit not only women’s reproductive health but overall human health.

Medicine involves evidence from research to guide its practice, but historically, clinical studies routinely excluded women for reasons including hormonal variability, potential harm to fetuses, and the belief that findings from research on men could be extrapolated to women^1^. These rationales and assumptions have hindered the study of how conditions like heart disease, diabetes, and Alzheimer’s Disease may affect women differently than men, as well as the study of conditions associated with women’s reproductive health, including adverse pregnancy outcomes, infertility, preterm birth (PTB), pre-eclampsia, recurrent pregnancy loss, endometriosis, adenomyosis, fibroids, and others^1,2^. In addition, representation of women in clinical trials has been traditionally lacking. Policy change is gradually resulting in improved representation of women in clinical trials^3^; nevertheless, research on women’s health conditions, particularly women’s reproductive health, remains underfunded and underprioritized^4–8^.

With advances in technology over time, ever-growing amounts of data have become available for basic science and translational research, such as molecular measurements—genomics, bulk and single-cell transcriptomics, proteomics, and also epidemiological and clinical data, including electronic medical records, clinical notes, images, and clinical trial data. Moreover, significantly greater computational power has allowed faster processing and analysis of large amounts of data. These advances provide tremendous opportunities to investigate a myriad of scientific questions in order to better understand the disease, discover novel diagnostics and therapeutics, make strides in precision medicine and more within many areas, including reproductive health sciences and women’s health, more broadly.

The advent of next-generation sequencing techniques and public data-sharing repositories have led to vast amounts of molecular data becoming widely available in recent years, enabling numerous studies and meta-analyses to gain insights into women’s health conditions (Fig. 1). For example, transcriptomics analyses have helped to enhance our understanding of endometriosis, a disorder affecting approximately 10% of women with pelvic pain and/or infertility whose diagnoses are made on average a decade after onset of their pain^9^. A study of eutopic endometrial transcriptomics data leveraging whole tissue deconvolution and single-cell RNA sequencing (scRNAseq) analytic techniques shed light into the immune as well as non-immune cells that most likely contribute to the pro-inflammatory nature associated with this disorder^10^. This endometrial expression data has been used to query the repository of drug expression data to identify and validate therapeutic candidates to treat endometriosis based on expression reversal. Fenoprofen, a non-steroidal anti-inflammatory drug (NSAID) rarely prescribed for endometriosis, was identified as a top candidate and tested in an animal model of endometriosis, which demonstrated its ability to successfully alleviate endometriosis-associated vaginal hyperalgesia^11^. With regard to PTB, a condition that affects ~10% of infants born each year and is the leading cause of infant morbidity and mortality worldwide^12^, a meta-analysis of maternal and fetal transcriptomics data found that immune signals are largely misregulated in women who end up delivering preterm with a reversed signal observed in babies^13^. This maternal expression signature was further used to query a repository of drug expression data to identify and validate therapeutic candidates to prevent PTB based on expression reversal. The study focused its validation efforts on lansoprazole, a proton-pump inhibitor, which has a strong reversal score and a good safety profile. Lansoprazole was tested in an animal inflammation model using LPS, which showed a significant increase in fetal viability compared with LPS treatment alone^14^.

**Fig. 1:**
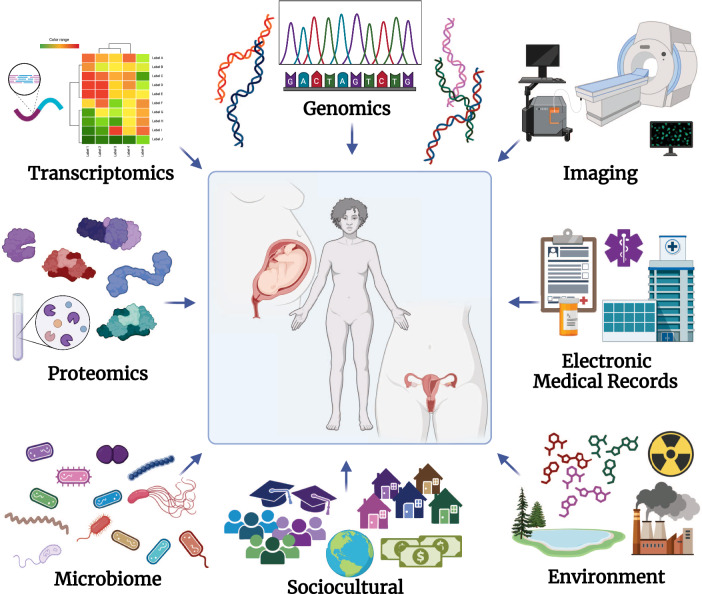
Data-driven approach to women’s health. This diagram showcases a number of types of data that can be leveraged to improve women’s health research, including genomics, transcriptomics, proteomics, microbiome, sociocultural, environmental exposures, EMRs and imaging. Created with BioRender.com.

There are a number of large-scale genetics studies to explore genomic loci associated with PTB, including a landmark study by Zhang et al., which consisted of 43,568 women of European ancestry using gestational duration as a continuous trait and term or preterm birth as a binary outcome^15^. In the discovery and replication data sets, four loci (EBF1, EEFSEC, AGTR2, and WNT4) were significantly associated with gestational duration, and functional analysis showed that an implicated variant in WNT4 alters the binding of the estrogen receptor. To probe the role of environmental exposures in pregnancy outcomes, an analysis of 590 matched maternal and cord blood samples (total 295 pairs) using non-targeted analysis (NTA) was able to examine the differences in chemical abundance between maternal and cord blood samples, hypothesizing which are able to cross the placenta^16^. This has inspired further large-scale integrative analyses of whole-genome sequences, RNAseq, and DNA methylation data to identify genomic variants and biomarker genes associated with PTB, such as Knijnenburg et al.’s study of 270 PTB and 521 control family trios^17^. In this study, they identified 72 candidate biomarker genes for very early PTB, associated with growth signaling and immunity-related pathways such as Notch1 and IFN-γ signaling. In addition, they identified PTB-associated genes RAB31 and RBPJ from all three data modalities.

In the microbiome space, there has been increased interest in the past few decades to characterize microbiome profiles across body sites in the context of pregnancy outcomes and identify specific microbes that can be associated with PTB. A meta-analysis of vaginal microbiome 16S rRNA sequencing data from five different studies confirmed that multiple known bacteria (e.g. *Atopobium* spp. and *Prevotella* spp.) and some novel organisms (*Clostridium sensu stricto* and *Olsenella*) are associated with PTB, and determined that diversity in the composition of the microbiome early during pregnancy was associated with PTB^18^. A study by Huang et al. integrated cross-sectional and longitudinal vaginal microbiome data from 12 previously published datasets and leveraged machine learning (ML) models to predict PTB from vaginal microbiome compositions, showing that the vaginal microbiome is a strong predictor of early PTB^19^.

A microbiome project led by our team applied the novel technique MaLiAmPi^20^ to aggregate and harmonize vaginal microbiome 16S rRNA sequencing data from a total of 11 different studies to see if PTB could be successfully predicted from microbiome data. The ability to harmonize 16S data across various studies marks a major contribution to the field, allowing researchers to collate larger datasets and ask more advanced questions about the effect of other factors, such as race and sampling time, on PTB. A crowdsourcing strategy in the form of a DREAM challenge invited the computational and scientific communities to develop and apply ML algorithms using this vaginal microbiome data to predict PTB. Model performance was assessed by challenge organizers using a held-out validation dataset not available to challenge participants. Over 300 individuals engaged in this challenge, and top-performing models from this challenge achieved excellent prediction performance with an area under the receiver operator characteristic (AUROC) curve of up to 0.87. Moreover, features such as alpha diversity, VALENCIA community state types, and microbial composition were found to be important for the top-performing models^21^. The above serves as a model for the translation of both new and publicly available molecular data into clinically relevant predictive models and a better understanding of the treatment and prevention of PTB. Moreover, studies are expanding beyond the associations between PTB and the vaginal microbiome: for example, DiGiulio et al. studied the dynamics of vaginal, distal gut, saliva, and tooth/gum microbiota throughout pregnancy in PTB vs. TB cohorts^22^. In addition, other cohorts and studies have been established, supplementing vaginal microbiome data with investigations of oral and gut microbiome changes, among other microbiomes, in PTB vs. TB pregnancies^23,24^. Advancements in genomic sequencing, such as whole-genome shotgun sequencing, allow scientists to go beyond ecological community characterization in PTB-associated microbiomes, exploring species-level genetic profiles and trends that may be associated with PTB. Liao et al. introduced the term “microdiversity” to describe genomic molecular diversity in their study that explored how evolutionary processes drive mutagenesis, nucleotide diversity, and antimicrobial resistance in specific species and in the vaginal microbial ecosystem^25^.

Beyond preterm birth, other reproductive health conditions have gained greater understanding from analyses of molecular data, including preeclampsia. The pregnancy-specific hypertensive disorders of preeclampsia, severe preeclampsia, and eclampsia affect ~6% of the US population and confer significant obstetric morbidity and mortality^26^. Efforts to find accurate diagnostic tools, preventative measures, and therapeutic treatments for preeclampsia have been elusive in part due to heterogeneity in its clinical presentation. Recently, computational approaches have made great strides in differentiating subtypes of preeclampsia using transcriptional analyses, effectively grouping the disorder into maternal, immunologic, and canonical groups based on gene expression^27^ as well as early (before 34 weeks gestation) vs. late (at or after 34 weeks gestation) onset preeclampsia^28^. Another recent study identified 946 unique differentially expressed genes in preeclampsia cited by prior microarray studies, defined the “ignorome”, which included 445 candidate genes that had never been experimentally explored, and utilized a biomedical knowledge graph to reveal 53 clinically relevant and biologically actionable mechanistic associations^29^. As technology has advanced from large chip microarray to bulk RNA sequencing and now to single-cell RNA sequencing, so too has our ability to develop greater granularity into the disorder. Most recently, immune profiling of peripheral blood mononuclear cells in preeclampsia and single-cell analyses of preeclampsia placentas offer mechanistic insight into individual cell-type contributions to the disorder^30^, lending hypotheses that can be tested in cell culture or animal models of preeclampsia. Taken together, the approaches demonstrate our ability to leverage molecular data to better understand the nature of this complicated condition.

A growing amount of clinical data has become available in this millennium since 2004, when the Bush administration outlined the Health Information Technology plan to assure Americans would have electronic health records to enable improved quality, affordability, and efficiency of health care^31^, and 2009 when the Obama administration prioritized and financially incentivized the transition from written to digital medical records as part of the Health Information Technology for Economic and Clinical Health (HITECH) Act^32^. Like written medical records, electronic medical records (EMR) capture clinical data on patient populations, including demographics, diagnosis codes, medication orders, and laboratory tests for patient care purposes. However, unlike their written counterpart, electronic records can be more readily de-identified and analyzed. Together with advanced computational approaches, researchers have been able to leverage billions of data points on millions of patients from sources such as EMRs, registries, and claims databases for clinical and translational research. Access to de-identified health records of individuals is currently limited and can be expensive to acquire through commercial sources. The availability of EMR data currently tends to be restricted to those who have affiliations with healthcare institutions, although there are efforts to have health records data available more broadly to those outside these settings^33^.

Analyses of EMRs have provided critical information about the incidence and prevalence of women’s health conditions and revealed associated diagnoses. With respect to endometriosis, EMR studies have delivered new insights across all these fronts. A decade-long retrospective cohort study completed using EMR found that the incidence rate of endometriosis declined from 2006 to 2015 while the frequency of chronic pelvic pain diagnoses increased, indicating a potential shift in diagnosis patterns or a relative change in the percentage of patients with endometriosis-associated conditions^34^. Another study investigating the validity of self-reported endometriosis by comparing it against medical record data found that self-reported diagnoses were reasonably accurate, ranging from 72% to 95% concordance across four international cohorts^35^. Towards phenotypic efforts, an analysis of medical record data from several hundred patients found a number of composite “pointers”, such as the onset of pain and menstrual symptoms within the same year, as significantly correlated with endometriosis years before an official diagnosis^36^. Moreover, when the COVID-19 global pandemic arose and dramatically changed clinical practice as well as the health of a population, researchers were able to promptly explore EMRs and investigate how the pandemic impacted women’s health. As pregnancy was a concern for being a risk factor for severe COVID-19, one cohort study analyzed EMRs of over 20,000 women from 82 healthcare centers across the U.S. during the first several months of the pandemic and found no difference in the risk of severe COVID-19 or mortality in pregnant versus non-pregnant women^37^. Another study explored pregnancy-related complications and maternal death in a healthcare database of 463 hospitals, with 849,544 women who were pregnant before the pandemic and 805,324 women who were pregnant during the pandemic. This study found that while the rates of several outcomes, including preterm birth, fetal deaths, and stillbirths, were unchanged, there were increases in maternal mortality during delivery hospitalization, pregnancy-related hypertensive disorders (i.e., gestational hypertension, pre-eclampsia, and eclampsia), and hemorrhage during the pandemic compared with before^38^. With regard to preventive care, the effect of COVID-19 stay-at-home orders on the rate of cervical cancer screening tests was explored in a large EMR database of nearly 1.5 million women that found that cervical cancer screening rate decreased significantly by ~80% during the lockdown compared to the year before the pandemic but returned to near baseline levels after the stay-at-home orders were lifted^39^. EMRs have also been leveraged to study the effects of various therapeutics in the context of pregnancy outcomes. For instance, a recent study explored the potential effects of serotonin selective reuptake inhibitor (SSRI) medications for the treatment of depression, which have been previously associated with PTB. This retrospective cohort study utilizing a sizeable primary care EHR dataset that included 216,070 deliveries of 176,866 patients over a 23-year period and a large-scale propensity score matching method that included all demographic and clinical covariates found that the risk of PTB is associated more so with depression rather than treatment with antidepressants^40^. While some previous observational studies found associations between exposure to antidepressants during pregnancy and increased risk of PTB^41,42^, the findings from this larger observational study could provide hope for those concerned about continuing antidepressant therapeutic regimen during pregnancy and motivate additional studies, particularly clinical trials, for further investigation. EMR data have also been used in efforts to predict outcomes of interest. One EMR-based study successfully leveraged the records of over 35,000 deliveries and found that when machine learning models were applied to this data, the models could not only successfully predict singleton PTB but outperform comparable models trained using only known PTB risk factors. Moreover, the prediction models were validated on a cohort of nearly 6000 deliveries from a different healthcare center with accuracy of the models maintained in this independent cohort^43^. Of course, there are many limitations to leveraging EMR data, including data missingness. Nonetheless, it is an incredible opportunity to leverage real-world patient data to impact disease diagnostics and therapeutics, as demonstrated by the examples above, especially in the area of women’s reproductive health.

Other sources of data have been investigated to better understand women’s reproductive health conditions, including patient registries and environmental exposure databases. Huang et al. linked the birth cohort file maintained by the California Office of Statewide Health Planning and Development across 1.8 million births and the CalEnviroScreen 3.0 dataset from California Communities Environmental Health Screening Tool and found an association between Pollution Burden, particulate matter ≤2.5 μm (PM2.5), and Drinking Water Scores and PTB. Additional findings suggest that certain drinking water contaminants, such as arsenic and nitrate, are associated with higher rates of PTB in California^44^.

There is great potential in the landscape of preterm birth, preeclampsia, endometriosis, and other women’s reproductive health disorders and the utility of molecular, clinical, and other data. Advanced computational models, machine learning approaches, and drug treatment identification enable researchers and clinicians to gain a better understanding and improve outcomes for these conditions. However, there are some limitations that should be recognized. Public data often suffers from incomplete and sometimes inaccurate meta-data. The populations that are captured in these datasets are often not representative of the general population. Therefore, we need data collection efforts to prioritize having an adequately broad representation of people from different backgrounds to reduce disparities and ensure that research findings and any resulting advances in healthcare practices benefit not just a subset of individuals but everyone^45,46^. Pregnant and lactating individuals should be specifically included in prospective studies and clinical trials, as our experience with the recent COVID-19 pandemic has attested to their exclusion in almost all vaccine and treatment trials and the subsequent gaps in data to provide counseling in pregnancy^47^. Other areas in which we lack data in pregnancy include immunologics utilized for autoimmune disease and organ transplant, as well as the best treatment for the pregnant person with significant medical comorbidities. As there is a lack of diversity not just among those who participate in and are represented in research but those who conduct research work, there should also be efforts to train, recruit, and support researchers from underrepresented backgrounds^48^.

It is also important to note that issues of data quality and bias, which must be tackled in all data-driven efforts, are equally relevant in women’s health research. In observational studies, selection bias (which itself has historically led to the exclusion of women in health research, as discussed in this perspective) can skew the composition of study populations along any number of demographic or clinical axes and profoundly affect the generalizability of findings^49^. Both clinical and experimental efforts can be prone to measurement errors, stemming from myriad causes such as mistakes in preparation or data collection and instrumentation flaws, which can then lead to deceptive conclusions^50^. Furthermore, confounding variables present a pervasive challenge throughout science, potentially masking the true effects of the variable of interest by being associated with both the exposure and the outcome^51^. In response to these challenges, we advocate for the continued improvement of research methods through the development and incorporation of standardized protocols^52^ and validation efforts^53^. Moreover, the adoption of transparent reporting practices, such as those laid out by CONSORT and STROBE initiatives^54^ or the Cell Press STAR Methods model^55^, will enhance reproducibility and underpin the integrity and credibility of data-driven findings in women’s reproductive health.

While advancements on the data collection and technical analysis methods fronts are essential to exploring concerns in women’s health, it is crucial to consider the impact of social determinants of health on patients’ presentations and clinical outcomes. For example, patients from low socioeconomic status who rely on Medicare or Medicaid or are under- or uninsured may not have reliable access to a physician to help manage gynecological conditions, causing adverse health outcomes^56^. In addition, medical racism is a culprit in the increased preterm birth rates in non-white women in the US^57^, and inequalities that can manifest in different forms—such as maternal stress and environmental exposure to toxins due to historical redlining—can contribute to preterm birth risk, as surveyed by epigenetic and gene-environment interaction studies^58^. Thus, it is crucial to adopt an intersectional approach to studying women’s health conditions, taking into account how cultural, socioeconomic, geographic, and racial disparity factors influence patients’ outcomes and healthcare experiences, which can inform a more holistic understanding of disease and contribute to improved approaches to care. A good first step would be to recruit larger, more diverse cohorts for studies to represent more realistic patient populations. Studies of women’s reproductive health should not focus solely on a person’s ability to have children or not but consider the individual holistically, including mental health and quality of life.

Challenges going forward will not necessarily be generating sufficient amounts of data for computational analyses but accurate phenotyping strategies, refining the analytical methods to gain greater biological insights, expanding on computational drug discovery opportunities for the advancement of therapeutics, finding ways that large language models and other new technological developments can enable discoveries, and bringing closer to reality the promise of precision medicine. Integrating and analyzing different types of -omics data to study women’s health conditions can provide revelations in causes of disease and targets for treatment^59^. Multi-omics approaches have resulted in greater insights into biological signals associated with term and preterm birth^60,61^ and could be increasingly leveraged to better understand pregnancy and other women’s health conditions. Moreover, digital twins can provide a data-driven way of monitoring, modeling, and managing conditions that can be tailored to an individual’s specific needs by integrating real-time data from various sources (e.g., clinical records, sensors, mobile health tracking applications, wearable devices) and artificial intelligence^62^. Digital twin technology could offer a transformative approach to women’s reproductive health, from identifying potential pregnancy complications early to managing endometriosis symptoms, finding optimal drugs and doses for treatments, and more. It is imperative, however, that we ensure discoveries from future research and technologies developed for women’s reproductive health do not widen the gap between those who are well-represented and privileged and those from under-represented and under-resourced backgrounds. Expanding on how we leverage molecular, clinical, sociocultural, and other data combined with robust computational integrative approaches for discoveries while we prioritize broader representation in studies will benefit not just women’s reproductive health but all areas of human health for everyone.
